# Cardiovascular risks before myocardial infarction differences between men and women

**DOI:** 10.1186/s12872-022-02555-3

**Published:** 2022-03-17

**Authors:** Alice Nyström, Susanne Strömberg, Karin Jansson, Åshild Olsen Faresjö, Tomas Faresjö

**Affiliations:** 1 Department of Health, Medicine and Care, General Practice, Linköping University, 581 83 Linkoping, Sweden; 2 Department of Cardiology, Vrinnevi Hospital, Norrköping, Region of Ostergotland Sweden

**Keywords:** Prodromal cardiac symptoms, Myocardial infarction, Gender, Risk factors for myocardial infarction

## Abstract

**Background:**

Prodromal cardiac symptoms are warning signals preceding cardiac disease. Previous studies have shown some gender differences in prodromal symptoms as well as established risk factors for MI. This study aims to map possible gender differences in social factors and established risk factors preceding myocardial infarction (MI).

**Methods:**

The study includes data of N = 213 middle-aged men and women, all diagnosed with myocardial infarction (ICD-10 I21.9) from the region of south-east Sweden. They answered a questionnaire at discharge from the cardiologic clinic and additional clinical data from medical records were merged from the National Swedeheart Register.

**Results:**

The dominant prodromal symptom for both sexes were experience of chest pain at the onset of MI. The major gender differences were that significantly more females (*p* = 0.015) had a hyperlipidemia diagnose. Females also reported to have experienced higher stress load the year preceding myocardial infarction with serious life events (*p* = 0.019), strained economy (*p* = 0.003), and reports of sadness/depression (*p* < 0.001). Females reported higher perceived stress load than men (*p* = 0.006). Men had higher systolic blood pressure than women at hospital admission and a higher systolic- and diastolic blood pressure at discharge.

**Conclusions:**

Influences of the social environment, such as serious life events, strained economy, depression, stress, and sleep deprivation were stronger as potential risk factors for myocardial infarction in women than among men. Of the traditional risk factors only, hyperlipidemia was more frequent among women. These findings could contribute to a deeper understanding of diagnostic differences between gender, as well as a more gender-oriented cardiovascular preventive work.

## Introduction

Prodromal cardiac symptoms are early warning signals preceding an acute cardiovascular event within days to months [1, 2]. At the onset of myocardial infarction (MI) both men and women present chest pain as predominant symptoms [3–5]. Prodromal symptoms of chest pain in acute coronary syndromes have been reported among up to 43% of the cases. However, women tend to report and experience more and not the same prodromal symptoms as men [6, 7]. Reports of sleep disturbances, anxiety, and fatigue and not prodromal chest pain are more frequent among women [6–8]. Patients under 65 years, regardless of gender, are more likely to report depression or major life events compared with elderly patients the year prior their MI [9]. In the acute stage, women are more likely to assign their prodromal symptoms to stress or anxiety, while men tend to assign their symptoms to cardiac disease [2].

The risk for MI can to a large extent be attributed to modifiable risk factors i.e. those that can be reduced or controlled with altered behavior. Besides heredity, also biological chronic stress and psychosocial stress contribute to the risk of MI [10–14]. Among established risk factors, certain risk factors, such as hypertension [14] and an adverse lipid profile [11], are more predominant than others on the risk of developing MI [11, 14]. Both genders have comparable risks for the association of MI with hyperlipidemia [13] and the prevalence is also similar in men and women [15]. The prevalence of hypertension is equal between the sexes globally [15, 16]. However, the impact of risk factors on each gender may differ. Smoking [13, 15, 17] and diabetes mellitus [15, 17] are more prevalent as risk factors in men than women. Smoking and obesity have the same risk on the sexes [13], while diabetes mellitus has a greater effect on females than males [13, 15, 17]. Men with hypertension, high BMI (body mass index) and type II diabetes have higher rates of MI than women with similar risk factors [18]. MI is more common in men than in women throughout life, but the gender differences in the risk MI decrease with age [19].

The overall aim in this study was to analyze if prodromal symptoms and social factors might differ between middle-aged men and women that suffers a myocardial infarction. We hypothesized that more men than women that had suffered an MI had established risk factors at the time of their myocardial infarction, and that chest pain was more prevalent in men than in women.

## Methodology

### Subjects

Data within the Stressheart study were collected from N = 213 patients, all diagnosed with myocardial infarction (ICD-10 I21.9) between 2017 and 2019 [10]. The cases were consecutively recruited during a three-year period from the cardiological clinics at three major hospitals in the region of south-east Sweden. The patients were invited to participate in the study at the time of discharge from the cardiology clinic, in general 2–3 days after the MI. Inclusion criteria were ST segment Elevation Myocardial Infarction (STEMI) or non-ST segment Elevation Myocardial Infarction (NSTEMI), and age up to 65 years. Exclusion criteria were not Swedish speaking. Overall, the exclusion and inclusions criteria in this study is identical to those applied in the Stressheart study [10].

### Data collection

At discharge from the cardiologic clinic at the hospital all patients were requested to fill in a questionnaire concerning social data and cardiovascular risk factors. The questionnaire included established risk factors and comorbidities such as hypertension, hyperlipidemia, diabetes mellitus, and smoking, but also questions of medical history (i.e. angina pectoris, heart failure, stroke, previous MI), medical treatment and potential heredity for MI or stroke. Questions about psychosocial factors (marital status, education, employment, social support) were also included in the questionnaire. Reliance for someone was defined as social support. Furthermore, participants were asked about various life events during the year that preceded MI, such as experience of serious life events, strained economy, and unemployment. Further, questions of depression, duration and quality of sleep and perceived stress levels. All scales and questions about psychosocial factors, serious life events, depression, sleep, and perceived stress were established and validated and derive from the large National Swedish CArdioPulmonary BioImage Study [22]. When the patients arrived at the hospital with suspected MI, clinical biomarkers on systolic blood pressure (SBP), diastolic blood pressure (DBP), heart rate (HR), high-density lipoproteins (HDL), low-density lipoproteins (LDL), cholesterol and triglycerides were collected. Risk prediction threshold values for lipids are based on the consensus-based recommendations from European Atherosclerosis Society and European Federation of Clinical Chemistry and Laboratory Medicine [20]. Height and weight i.e. BMI, was documented by research nurses.

Data for all the participants were merged with clinical data from medical records in the Swedish National Register of Myocardial Infarction (The Swedeheart Register). The study was approved by the Regional Ethical Review Board in Linköping, Sweden (Dnr 2016-79-31, Dnr 2016-453-32, Dnr 2017-106-32).

### Statistical analysis

Descriptive statistics were performed with gender as the independent variable. Variables with normal distribution were presented as mean and standard deviation (SD). Variables with skewed distribution were presented as the median and interquartile range (Q1–Q3). Significance of difference between gender was assessed with T-test for normally distributed variables, and with Mann Whitney U for skewed variables. The significance of differences between categorical variables and gender was assessed by using Chi-square tests and Mann Whitney U. Statistical significance was set at *p* ≤ 0.05 for all analyses. All statistical analyses were assessed in IBM Statistical package SPSS for social sciences version 27 (SPSS, Chicago, Illinois, USA).

## Results

All the N = 213 participants in this study were diagnosed with MI of which 73% were males and 27% females. Myocardial Infarction with STEMI was diagnosed for 56.8% of men and 60.7% of females and 43.2% of men and 39.3% of females had NSTEMI. There was no significant difference of infarction type between the sexes (*p* = 0.618). No age difference between the sexes (*p* = 0.431), mean age for males was 58 ± 6 years and for females 57 ± 7 years. A vast majority of both males and females debuted their onset of MI with chest pain (males 93.9% vs females 94.4%) and a smaller proportion with dyspnea or other (males 6.1% vs females 5.6%). There was no significant difference in symptoms at MI onset between the sexes (*p* = 0.885) (data not shown).

Demographics for males and females in the study are presented in Table 1. No differences in marital status between the sexes found, neither the access to a supportive person. Highest level of education did not differ significantly between the sexes (*p* = 0.794), however, employment did. More males were working 79.5% vs. 62.5% among females (*p* = 0.021). Unemployment rates was significantly higher (*p* = 0.008) among females than among men (8.3% vs 0.8%) and females were more frequent (*p* = 0.053) on sick leave over the last three months before MI (10.4% vs 3.1%), see Table 1.

**Table 1:** Demographics for males and females with MI

	Males (N = 156)	Females (N = 57)	*p* value
	n (%)	n (%)	
Single civil status	34 (21.8)	13 (22.8)	0.875
Partner	121 (78.1)	44 (77.2)	0.892
Social support	145 (94.2)	51 (89.5)	0.240
Highest level of education			
Elementary school	36 (23.1)	14 (24.6)	0.794
Gymnasium/high school	86 (55.1)	33 (57.9)	
University or similar	34 (21.8)	10 (17.5)	
Employment			
Working	101 (79.5)	30 (62.5)	**0.021**
Unemployed	1 (0.8)	4 (8.3)	**0.008**
Retired	26 (20.5)	13 (27.1)	0.348
On sick leave > 3 months	4 (3.1)	5 (10.4)	**0.053**

Statistical significance was set at *p* ≤ 0.05 and marked as bold

During the year preceding the MI, more females than males had experienced various serious life events (Table 2). Significant differences between the sexes were evident for serious life events in general (*p* = 0.019), strained economy (*p* = 0.003) and sadness/depression for two weeks or more (*p* < 0.001), see Table 2.

**Table 2:** Experience of serious life events past year for males and females with MI

	Males (N = 156)	Females (N = 57)	*p* value
	n (%)	n (%)	
*Serious life events past year*			
Serious life event	61 (39.9)	33 (57.9)	**0.019**
Death/accident among friends/family	40 (26.1)	22 (39.3)	0.065
Divorce/ separation	3 (2.4)	3 (6.4)	0.209
Severe illness in the family	13 (10.5)	10 (21.3)	0.065
Economic crisis	4 (3.2)	3 (6.4)	0.354
Unemployment	2 (1.6)	2 (4.3)	0.309
Strained economy	6 (4.7)	9 (18.8)	**0.003**
Sadness/ depression ≥2 weeks	40 (26.5)	30 (53.6)	** < 0.001**

Statistical significance was set at *p* ≤ 0.05 and marked as bold

More men than women had a previous medical history of cardiovascular diseases like MI, angina pectoris, heart failure, stroke, and heredity of stroke, but these tendencies were not statistically significant. There was neither any significant difference between men and women observed for prevalence of diabetes, hypertension, or heredity for MI, obesity, BMI (Table 3). Equal proportions of men and women had hypertension diagnosed before the onset of MI and equal proportions among these had antihypertensive treatment (60% vs 57.1%, *p* = 0.795). Slightly more women than men had diabetes mellitus (32.1% vs 22.9%), but not statistically significant (*p* = 0.184). More women than men were both current smokers (females 59.6%, males 45.5%, *p* = 0.068), and current and former smokers (females 91.2%, males 81.4%, *p* = 0.083). A diagnose of hyperlipidemia was more prevalent in females than in males (35.4% vs 18.1%, *p* = 0.015). However, there were no significant differences in the levels of cholesterol, triglycerides, LDL, HDL, or LDL/HDL-ratio between the sexes (see Table 3). Among patients with hyperlipidemia, 47.1% of females and 52.2% of males had lipid lowering treatment (*p* = 0.749).

**Table 3:** Previous cardiovascular diseases and established risk factors for males and females with MI

	Males (N = 156)	Females (N = 57)	*p* value
	n (%)	n (%)	
*Previous cardiovascular diseases*			
Myocardial infarction	36 (26.1)	11 (20.8)	0.444
Angina pectoris	16 (12.6)	3 (6.3)	0.228
Heart failure	4 (3.1)	1 (2.1)	0.706
Stroke	8 (6.3)	2 (4.2)	0.588
Heredity stroke	41 (37.3)	14 (32.6)	0.585
*Established risk factors*			
Diabetes (type 1 and type 2)	35 (22.9)	17 (32.1)	0.184
Hypertension	80 (54.1)	29 (50.9)	0.683
Heredity myocardial infarction	60 (51.7)	26 (59.1)	0.404
Current smoker	71 (45.5)	34 (59.6)	0.068
Current or former smoker	127 (81.4)	52 (91.2)	0.083
Obesity (BMI ≥ 30)	47 (30.1)	15 (26.8)	0.637
BMI [kg/m^2^] *Mean (SD)*	28 (4)	26.8 (5.5)	0.135
Hyperlipidemia	23 (18.1)	17 (35.4)	**0.015**
Cholesterol [mmol/L] *Median (IQR)*	5.3 (4.3–6.1)	5.4 (4.3–6.1)	0.866
Triglycerides [mmol/L] *Median (IQR)*	1.7 (1.3–2.9)	1.8 (1.2–2.8)	0.833
LDL [mmol/L] *Mean (SD)*	2.9 (1.2)	3.1 (1.2)	0.256
HDL [mmol/L] *Median (IQR)*	1.2 (0.9–1.4)	1.1 (0.9–1.3)	0.262
LDL/HDL-ratio *Mean (SD)*	2.7 (1.1)	3 (1.1)	0.103

Statistical significance was set at *p* ≤ 0.05 and marked as bold

**Fig. 1:**
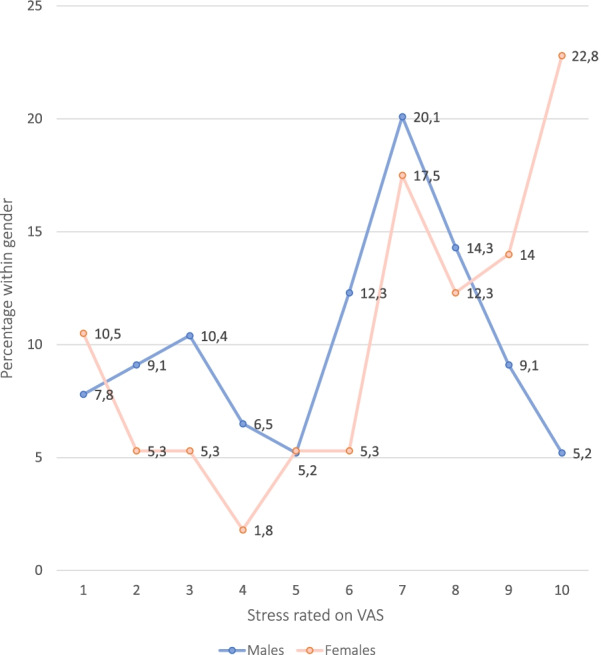
Assessments of stress levels on a visual analog scale from 1 (low) to 10 (high) for males and females with MI

Females tend to have higher perceived stress load than men (Fig. 1). The median rating for females was 7 (IQR = 5–9) and for men 6 (IQR = 3–8) (*p* = 0.006). Equal proportions of the sexes (5.2% of males, 5.3% of females) estimated their stress level as 5. More women than men rated their stress at the highest level (9–10), see Fig. 1.

**Fig. 2:**
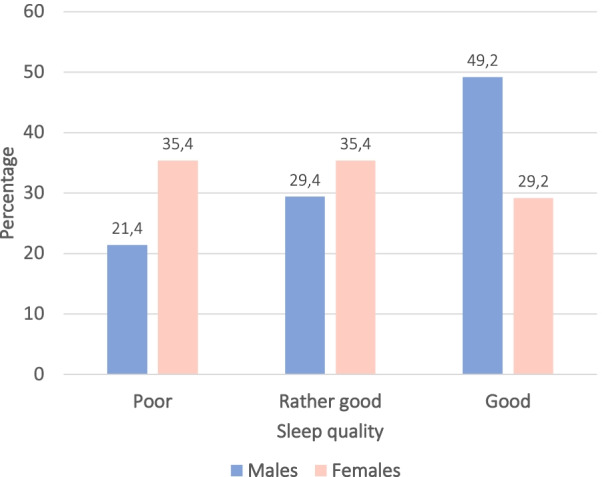
Estimated sleep quality among males and females with MI

Men and women evaluated their sleep quality differently (*p* = 0.043), see Fig. 2. More females than males perceived their sleep quality as poor (35.4% vs 21.4%) and contrary, more males than females estimated their sleep quality as good (49.2% vs 29.2%). Further, women tend to have a shorter sleep duration than men. The median sleep hours for males were 7 h (IQR = 6–8) and for females 6 h (IQR = 5–7) (*p* = 0.02).

Females had a higher Systolic Blood Pressure (SBP) at hospital admission compared to men 153 mmHg (IQR 139–168) versus 146 mmHg (IQR 124–162), *p* = 0.046. Further, men had a higher SBP the day of discharge compared to women 127 mmHg (SD = 16) versus 118 mmHg (SD = 17) *p* < 0.001). At admission there was no significant difference in Diastolic Blood Pressure (DBP) between the sexes, males 90 mmHg (IQR 80–105) versus females 96 mmHg (IQR = 85–105) *p* = 0.227). However, men had a higher DBP than women at discharge 80 mmHg (IQR 74–85) versus 74 mmHg (IQR 64–85) *p* = 0.001). The Heart Rate (HR) in men was 78 (SD = 19) at admission compared to 76 (SD = 18) beats per minute in females (*p* = 0.684). At discharge the HR in men was 71 (SD = 11), compared to 75 (SD = 13) beats per minute in females (*p* = 0.035).

## Discussion

### The main findings

The main findings in this study of middle-aged men and women that had suffered a myocardial infarction was that more women than men had hyperlipidemia diagnosed at the cardiac event and that more females had experienced higher stress load including perceived stress, serious life events, strained economy, and depression the year preceding their MI. Females also estimated poorer sleep quality and shorter sleep duration than men. More females than men were unemployed or on sick leave. Blood pressure and heart rate differed significantly between gender at discharge from the hospital. Most of both genders experienced chest pain as a prodromal symptom at the onset of MI. We hypothesized that more men than women had established risk factors at the time of their MI. But in contrast, significant differences between gender were only observed for hyperlipidemia that were on the contrary significantly more frequent diagnosed among females.

### Lipid profiles

In our study 35.4% of women and 18.1% of men had diagnosed hyperlipidemia before the MI (*p* = 0.015). However, there was no statistical significance between the sexes in lipid values. Participants with hyperlipidemia could have both been diagnosed and medicated, which subsequently affects the level of the lipid values and thereby gender differences. As the mean age for women in our study was 57 ± 7 years, this indicates that the majority were postmenopausal. Women have a more favorable lipid profile before the menopausal transition, after which a shift takes place and men have a better lipid profile in older ages [15]. This explanatory model of a lipid shift in connection with the menopausal transition is a reasonable partial explanation of why more women than men had hyperlipidemia in our study. However, it is with caution that an explanatory model for lipid levels between sexes in a normal population translates to our study participants that all had an MI.

In “the VIRGO study”, which has a similar methodology in terms of study design, there was an opposite relationship between gender, where 72.2% of men and 66.4% of women had hyperlipidemia [2]. In the VIRGO study the average age of women was 47 years [2]. Thus, it can be assumed that the reverse relationship depends on that a much smaller proportion had undergone the menopausal transition. The difference could also be explained by the higher prevalence of obesity in females in the VIRGO study compared to our study (55.3% vs 26.8%). In the VIRGO study [2] the percentage points for hyperlipidemia in both sexes were markedly higher than in our study. This is to be expected as hyperlipidemia is a major public health problem in the US [21], and because of lifestyle changes in Sweden that entailed reduced cholesterol levels [22, 23].

### Smoking as risk factor

Remarkably 59.6% of the females in our population with MI-cases were current smokers and 92% former smoker. This reflects that smoking has a major impact on women's cardiovascular risk, which previously been confirmed in a study that stated that the impact of smoking on the risk of CVD is greater in females than in males [15]. This study is only based on patients with MI and no healthy control group was used in the analyses. Thus, there is no statistical basis for further reflecting on the impact of smoking on the risk of MI, for each gender. In this study we did not demonstrate any significant gender differences in smoking, but the statistical analysis shows a tendency that more middle-aged women than men with MI smoke or have smoked. This suggests that smoking might possibly have a greater impact on women than men in these age-groups.

### Stress as risk factor

Our results with significant gender differences for strained economy, sadness/ depression, and serious life events, shows that these factors appear to be stronger potential risk cardiovascular factors for women than for men. Previous studies have shown consistent results, for example, that more women than men have experienced stress the year before MI, such as depression [2, 9] and sleep disorders [9]. Our study demonstrated that women had poorer sleep quality and a shorter subjective sleep duration than men. Whether this is a consequence of stress, or an independent factor is not possible to assess. Previous studies have shown that a higher proportion of those with disturbed sleep has an adverse cardiac risk profile [24]. This suggests that the gender differences in sleep, shown in our study, have some association with the risk of MI. In our study population, females had a higher stress load than men. This differs from a previous published result, that did not show any significant difference for stress level between gender [9]. Stress was not stated likewise in the studies, which may affect the difference in results as our study used a visual analog scale and the compared study [9] used several response alternatives.

The demonstrated statistical significance between the sexes in SBP at hospital admission, and for SBP, DBP, and HR at discharge is not of clinical relevance due to small differences. Based on risk prediction thresholds it can be observed that the measured value (described as mean or median) for triglycerides was within acceptable range, but LDL was higher than clinically desirable for women, and HDL-cholesterol lower than clinically desirable. For men, the median value for HDL and triglycerides, as well as the mean value for LDL was within an acceptable range.

### Strengths and limitations

A strength in this study is that all participants had a validated diagnose of myocardial infarction and that other clinical data was merged with data from the National Swedeheart Register. This has resulted in an opportunity to map factors, at an individual level, that were present before the onset of MI and clinical measurement values present at the time of the disease. We reduced the possible risk of recall bias by merging the variables that were comparable between the Stressheart database and the National Swedeheart Register, which were diabetes, hypertension, and smoking (current and current or former). As we chose to focus on gender differences in a middle-aged population with MI, the results can only be considered as a mapping of possible risk factors, whose significance for the risk of MI is unproven. The basic design in this study, where we compare men and women that all had suffered a myocardial infarction, is that we primary compare their social and general conditions before the onset of MI. This design means that we don´t have included a healthy control group, which from some aspects could be a limitation. A general limitation in this study could be the relatively limited sample size. However, the sample size was sufficient for our statistical analysis, also according to power calculations. Another limitation is the skewed sex distribution, but this reflects quite well the uneven sex distribution of men and women in this age-group that suffers a myocardial infarction.

## Conclusion

Influences of the social environment, such as serious life events, strained finances, depression, stress, and sleep deprivation were stronger as potential risk factors for myocardial infarction in women than among men. Of the traditional risk factors only, hyperlipidemia was more frequent among women. These findings could contribute to a deeper understanding of diagnostic differences between gender, as well as a more gender-oriented cardiovascular preventive work. A challenge for future studies is to have a more thoroughly and deeper analysis of potential social and psychosocial factors preceding cardiac events especially for middle-aged women.

## Data Availability

The dataset presented in this article is available only upon reasonable request, since it contains confidential information. Requests to access the dataset should be directed to the corresponding author (tomas.faresjo@liu.se).
